# Chondroitin Sulfate/Dermatan Sulfate-Protein Interactions and Their Biological Functions in Human Diseases: Implications and Analytical Tools

**DOI:** 10.3389/fcell.2021.693563

**Published:** 2021-08-06

**Authors:** Bin Zhang, Lianli Chi

**Affiliations:** National Glycoengineering Research Center, Shandong University, Qingdao, China

**Keywords:** chondroitin sulfate, dermatan sulfate, protein, interaction, human disease, analytical methods

## Abstract

Chondroitin sulfate (CS) and dermatan sulfate (DS) are linear anionic polysaccharides that are widely present on the cell surface and in the cell matrix and connective tissue. CS and DS chains are usually attached to core proteins and are present in the form of proteoglycans (PGs). They not only are important structural substances but also bind to a variety of cytokines, growth factors, cell surface receptors, adhesion molecules, enzymes and fibrillary glycoproteins to execute series of important biological functions. CS and DS exhibit variable sulfation patterns and different sequence arrangements, and their molecular weights also vary within a large range, increasing the structural complexity and diversity of CS/DS. The structure-function relationship of CS/DS PGs directly and indirectly involves them in a variety of physiological and pathological processes. Accumulating evidence suggests that CS/DS serves as an important cofactor for many cell behaviors. Understanding the molecular basis of these interactions helps to elucidate the occurrence and development of various diseases and the development of new therapeutic approaches. The present article reviews the physiological and pathological processes in which CS and DS participate through their interactions with different proteins. Moreover, classic and emerging glycosaminoglycan (GAG)-protein interaction analysis tools and their applications in CS/DS-protein characterization are also discussed.

## Introduction

**FIGURE 1:**
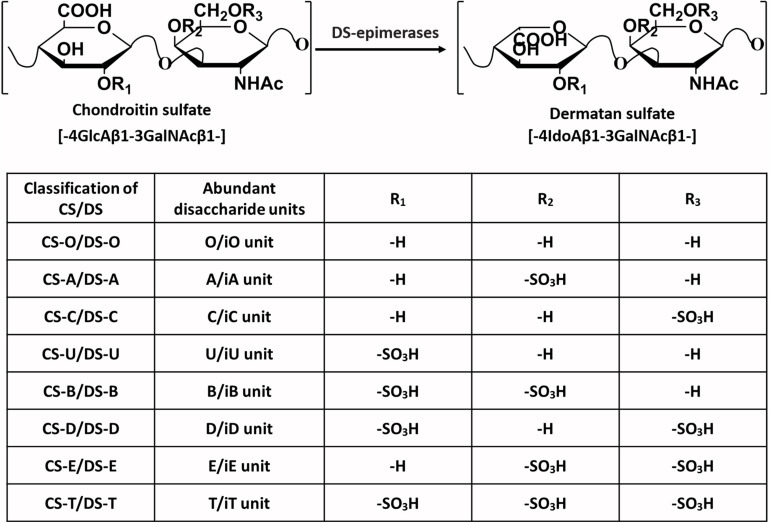
The typical disaccharide units present in the backbone of the CS/DS chain. The major types of disaccharide units and sulfate group substitution positions that frequently exist in the CS/DS chains of different classifications are given.

Chondroitin sulfate (CS) and dermatan sulfate (DS) are acidic linear anionic polysaccharides in the glycosaminoglycan (GAG) family. CS is composed of repeated disaccharide units [→4GlcAβ1→3 GalNAcβ1→], where GlcA and GalNAc refer to D-glucuronic acid and N-acetylgalactosamine (Mikami and Kitagawa, 2013). The difference between the DS chain and CS chain is that the GlcA is epimerized to iduronic acid (IdoA) in the DS, and the CS and DS structures are usually found in a single CS-DS hybrid polysaccharide chain (Sugahara et al., 2003). The structure of CS/DS seems simple, but the hydroxyl groups at the C4 and C6 positions of GalNAc and the C2 position of GlcA/IdoA residues may undergo sulfation modification, thus yielding huge structural variations (Mizumoto et al., 2015). According to the number and positions of sulfate groups, the disaccharide units can be divided into different types, as shown in Figure 1. A single polysaccharide chain generally contains different disaccharide units, and CS and DS are usually classified by their most abundant disaccharide units. For example, the CS-A chain contains not only the dominant A unit but also relatively small amounts of C units and O units (Wang W. et al., 2020). The diversity of CS/DS structures leads to different biological functions, but it also brings great challenges to the study of corresponding structure-function relationships.

Like most GAGs, CS and DS are located in the animal extracellular matrix (ECM), on the cell surface or associated with the plasma membrane (Li et al., 2019). Therefore, they seem to be strategically in a superior position to control various important processes that occur at the cell-tissue interface. CS/DS chains are covalently bound to the core protein to form CS/DS proteoglycans (CS/DSPG), which participate in the regulation of the extracellular environment and many biological and pathophysiological activities (Malavaki et al., 2008). In recent decades, increasing evidence has shown that CS/DS is involved in tumor occurrence and metastasis (Knutson et al., 1996; Tang et al., 2018), nervous system development (Avram et al., 2014; Shida et al., 2019), virus adsorption and infection (Aquino and Park, 2016; Yang et al., 2016), atherosclerosis and other diseases (Pomin, 2015; Stabler et al., 2017; Scuruchi et al., 2020). The CS/DS chain performs these functions by interacting with various target proteins, such as various growth factors, fibroblast growth factor (FGF), hepatocyte growth factor (HGF), pluripotent protein (PTN), cell surface receptor and intercellular adhesion factor (Pudelko et al., 2019).

**FIGURE 2:**
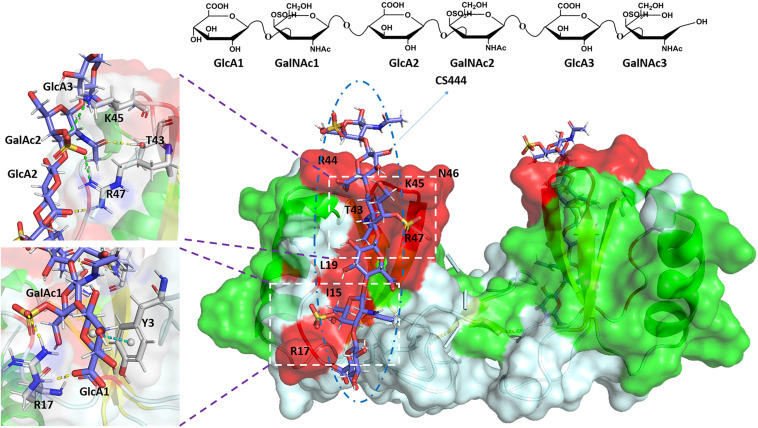
Complex of CCL5 dimer and CS444. In the cartoon and surface hybrid model, the CS combined area is displayed in red. In the enlarged image, other non-covalent interactions are shown. The yellow dashed line represents hydrogen bonds, the green dashed line represents ionic bonds, and the blue dashed line represents ring-stacking interactions. The figure was prepared using the PDB file originally reported in the reference (Deshauer et al., 2015).

The charge density and the position of sulfation on the CS/DS chains are the main factors that affect their ability to interact with target proteins, while a minimal chain length is usually required for binding (Bao et al., 2005; Yamaguchi et al., 2006). It was initially believed that the GAG-protein interaction was solely due to charge-charge interaction. On this basis, a number of consensus sequences on GAG binding proteins were found, including XBBXBX, XBBBXXBX, and XBBBXXBBBXXBBX, where B is a basic amino acid residue and X is a hydrophilic residue (Cardin and Weintraub, 1989; Sobel et al., 1992). In subsequent studies, it was found that non-ionic interactions (hydrogen bonds, hydrophobic interactions) and the secondary, tertiary structure or spatial distribution of basic amino acid residues may also have an important impact on the interaction between these two biomolecules (Margalit et al., 1993; Bae et al., 1994; Hileman et al., 1998). The CS-protein binding is much more complex than the simple charge to charge interaction has also been supported by that the CS chains bearing the same charge to mass ratio are frequently observed to exhibit difference in binding specificity and affinity. For examples, nephronectin bound strongly to CS-E, but failed to bind to CS-D, although both CS-D and CS-E are carrying two sulfate groups per disaccharide unit (Sato et al., 2013); The FGF family also showed distinct affinities when binds to CS-D and CS-E, as the FGF3, 6, 8, and 22 bound strongly to CS-E, while the FGF5 bound moderately to CS-D (Asada et al., 2009). The binding between the CS and C-C motif chemokine ligand 5 (CCL5) was presented in Figure 2 as an example. In addition to the basic BBXB binding motif, within the binding area of CS there are also hydrogen bonds, ionic bonds, and ring-stacking interaction that participate in the combination of the complex (Deshauer et al., 2015).

Characterization of the CS/DS-protein complex is of great significance for the design of more specifically targeted protein/GAG analogs and the subsequent development of more effective therapeutic drugs with fewer side effects. However, the structural motifs and interaction mechanisms between CS/DS and proteins are not fully understood due to the heterogeneity and complexity of CS/DS structures. The rapid development of analytical methods, including affinity approaches, mass spectrometry (MS), nuclear magnetic resonance (NMR), and computational approaches, makes it possible to study the GAG-protein complex with higher resolution, which will help in understanding the interaction mechanisms between CS/DS and various protein ligands and in the development of new treatment methods.

This review focuses on the interactions between CS/DS and proteins and their roles in human diseases. In addition, some classic and emerging GAG-protein interaction analysis tools and their applications in CS/DS-protein characterization are also discussed.

## Human Diseases Related to CS/DS-Protein Interactions

**FIGURE 3:**
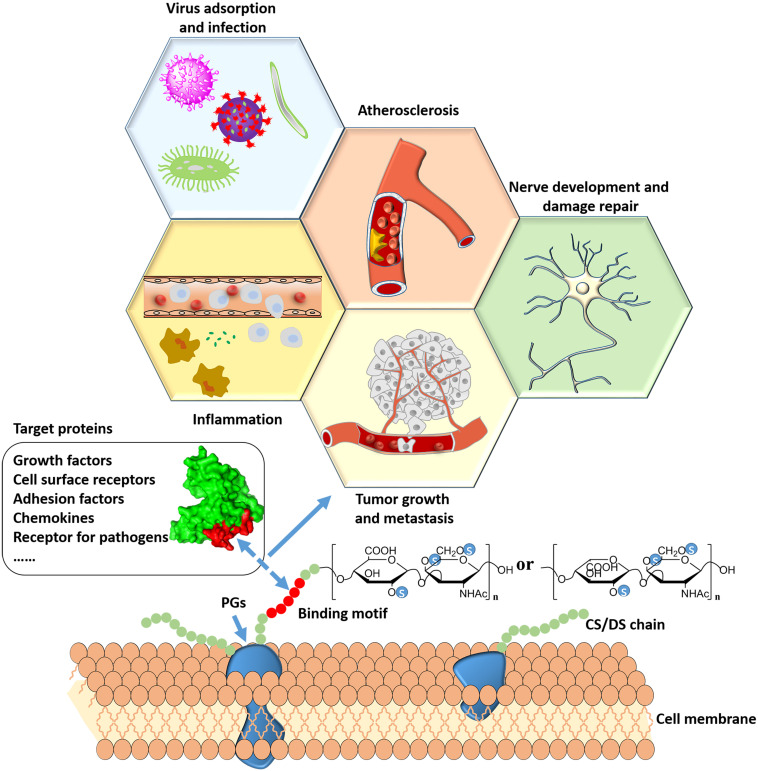
Major diseases that are related to interactions between CS/DS and proteins.

In the human body, CS/DS plays an important role in the physiological and pathological processes by interacting with a large number of proteins. The development of certain diseases is usually related to the imbalance of CS/DSPGs biosynthesis and changes in CS/DS chain structure. The major diseases that are related to the interaction between CS/DS and proteins are presented in Figure 3.

### Tumor Growth and Metastasis

Tumor-associated glycans and PGs play an important role in promoting the invasion and metastasis of malignant cells, participating in cell-cell and cell-ECM interactions, and promoting the adhesion and migration of tumor cells. In many tumor tissues, changes in the production level of PGs and the structure of GAG have been observed. Among PGs, the important role of HSPG in tumor adhesion, invasion, migration, proliferation and angiogenesis has been recognized for a long time (Afratis et al., 2012). In recent years, increasing evidence has shown that in addition to HSPGs, CS/DSPGs are also important regulatory molecules that affect tumor cell functions and phenotypes (Fuster and Esko, 2005). Versican and decorin are the main CSPGs that are overexpressed in the ECM of many malignant tumors, such as osteosarcoma, breast cancer, testicular cancer, colon cancer and pancreatic cancer (Labropoulou et al., 2006; Theocharis et al., 2006; Skandalis et al., 2011). Versican has been shown to induce the secretion of inflammatory factors by macrophages by activating the TLR2:TL6 complex, which can change the inflammatory microenvironment of tumors, thereby promoting tumor cell metastasis (Kim et al., 2009). Tumor cells spread to other organs through platelets. Studies have shown that CS/DS on the surface of breast cancer cells interacts with p-selectin ligands on endothelial cells and activated platelets, thereby promoting the spread of tumor cells (Cooney et al., 2011). The DS chain of endocan expressed in the capillaries of tumor tissues can bind to and activate HGF to promote tumor cell movement and proliferation (Delehedde et al., 2013).

The different sulfation patterns of the disaccharide repeating unit endow CS with different polyanionic properties, making CS have different biological activities. For example, the 6-*O*-sulfation of CS exhibited the dual role in the development, progression and metastasis of cancer. The monosulfated substitution provided interaction surface for CS and influenced its binding to various cytokines and growth factors, cell surface receptors and other important proteins, which resulted in the complex role of CS/DS in tumor (Pudelko et al., 2019). In the case of disulfated CS, studies have shown that CS-E played a critical role in the tumorigenesis process. The ECM of ovarian cancer and adenocarcinoma cells can secrete excessive amounts of CS-E, which can combine with vascular endothelial growth factor (VEGF) to regulate VEGF signal transduction and CD44 molecular hydrolysis. Among them, VEGF can regulate the angiogenesis of tumor tissues, and the hydrolysis of CD44 molecules is beneficial to the metastasis of tumor cells, thereby enhancing the ability of tumor cells to metastasize (ten Dam et al., 2007; Sugahara et al., 2008). CS-E undergoes stronger binding to P-selectin on the surface of tumor cells and mediates signal transduction in the process of tumor metastasis. In addition, CS-E plays an important role in the migration of Lewis lung cancer cells by interacting with advanced glycation end products (RAGE) (Mizumoto et al., 2012). The specific changes in CS structures during tumorigenesis and development indicate their importance as potential biomarkers for cancer occurrence and for development and as therapeutic targets.

### Nerve Development and Damage Repair

CS is the GAG with the highest content in the matrix of the central nervous system (CNS), as CSPG accounts for about 20% of the total ECM of the CNS (Djerbal et al., 2017). Notably, many studies have shown that CSPG has a two-way function in the development of the nervous system. On the one hand, CSPG is involved in almost all processes of nervous system cell proliferation and differentiation, nerve migration, axon growth and synapse formation and stability, which is essential for the orderly development and maintenance of the nervous system (Mencio et al., 2021). The neural stem cell niche of embryonic and adult forebrain is rich in CS, which is essential for the development and maintenance of neural stem/progenitor cells mediated by FGF-2 (Sirko et al., 2010; Bian et al., 2011). Additionally, CSPGs can be combined with a variety of growth factors, such as midkine (MK), pleiotrophin (PTN), brain-derived neurotrophic factor (BDNF) and other neurotrophic factor family members, to regulate growth signal pathways and promote neuron growth (Rogers et al., 2011; Miller and Hsieh-Wilson, 2015). The CS on CSPGs can also be used as a guide for growth cones and promote the formation of neuron boundaries in the developing CNS (Dyck and Karimi-Abdolrezaee, 2015). On the other hand, CSPG is also involved in inhibiting the plasticity and regeneration of the adult CNS. CSPG is significantly upregulated in glial scar areas around CNS injuries such as those due to trauma and stroke, inhibits neuron growth and axon regeneration by binding to the receptor protein tyrosine phosphatases protein tyrosine phosphatase σ (RPTPσ) and LAR and to Nogo receptors (NgR1 and NgR3) (Brown et al., 2012; Griffith et al., 2017). In addition, neurocan (Ncan), as a CNS-specific CSPG, is involved in visual processing and top-down cognitive functions. Changes in Ncan are potential risk factors for bipolar disorder and schizophrenia (Schultz et al., 2014). Brevican (Bcan) is another specific CSPG of CNS. It not only participates in neuronal plasticity as a structural component, but is also considered to be related to CNS damage and Alzheimer’s disease (Jang et al., 2020). The above studies show that the roles of CSPGs in the growth and development of the nervous system are extensive, complex, sometimes contradictory, and, of course, essential.

Currently, some *in vitro* studies have proven the specificity of the sulfation mode for some of the above processes. *In vitro* experiments have shown that CS-E and CS-D, which are rich in highly sulfated modifications, are very important for regulating the functions of CSPGs. They can interact with growth factors and inhibitory cell surface receptors (Miller and Hsieh-Wilson, 2015). For example, CS-D and CS-E can be combined with the growth factors MK and PTN to promote neuron growth, but CS-A and CS-C cannot (Deepa et al., 2002; Maeda et al., 2003). Moreover, CS-E can also participate in the inhibition of nerve damage repair by interacting with the cell surface receptors RPTPσ and NgRs (NgR1 and NgR3) (Dickendesher et al., 2012; Griffith et al., 2017). Therefore, further study of CS sulfation patterns in the nervous system and their interacting proteins will provide a molecular basis for the development of new therapies to promote neuronal growth and regeneration and CNS plasticity.

### Virus Adsorption and Infection

Most pathogens need to use GAG on the cell surface to promote their attachment and infect host cells, transfer from one cell to another, and evade host defense mechanisms. Many pathogenic microorganisms, such as viruses (e.g., dengue virus; Avirutnan et al., 2007), herpes simplex virus (HSV) (Uyama et al., 2006), vaccinia virus, and respiratory syncytial virus (RSV) (Klenk and Roberts, 2002), bacteria (e.g., *Borrelia burgdorferi*; Srinoulprasert et al., 2006) and fungi (e.g., Penicillium), can express proteins that bind to CS/DS on the cell surface, thereby promoting the infection of host cells.

The adsorption and invasion of many pathogens rely on CS/DS motifs with specific sulfation modification patterns on the host cell surface. The non-structural protein NS1 secreted by the dengue virus can bind to HS and CS-E on the surface of host cells and mediate the accumulation of NS1 on microvascular endothelial cells, leading to immune-mediated vascular damage and leakage (Avirutnan et al., 2007). Plasmodium invades erythrocytes and secretes the VAR2CSA protein. VAR2CSA can specifically bind to CS-A on the surface of vascular endothelial cells to make infected erythrocytes adhere to the blood vessel wall and cause falciparum malaria (Clausen et al., 2012; Rieger et al., 2015). HSV is a typical representative herpesvirus that can effectively bind to GAG and other receptors on the cell surface to infect host cells. Among HSV receptors, HS has been extensively studied, but there are also studies showing that CS-E is also important (Uyama et al., 2006). In addition, during the infection process of Penicillium, its conidia can adhere to CS-B and HS on the surface of epithelial cells to exert an infectious effect (Srinoulprasert et al., 2006). In short, these observations emphasize the biological significance of the interactions between CS/DS and pathogens in infectious diseases, which provides a theoretical basis for the development of antiviral drugs based on CS/DS.

### Atherosclerosis

Atherosclerosis (ATH) is a chronic, dynamic and evolving process that involves changes in the morphology and structure of arterial vessels, leading to the formation of atherosclerotic plaques, which ultimately leads to myocardial infarction or stroke (Scuruchi et al., 2020). PGs are considered essential molecules for maintaining vascular homeostasis, and changes in their regulation are key factors in the induction and development of ATH. The most representative vascular PGs are CSPGs and DS-containing DSPGs, such as versican, biglycan, and decorin. The deposition of low-density lipoprotein (LDL) in the arteries has been considered the initiating factor in the development of atherosclerosis. CSPGs significantly increase in early atherosclerotic lesions and play an important role in lipid retention, modification and final accumulation (Karangelis et al., 2010). Biglycan is considered the most likely to participate in the retention of lipids in the vascular intima because it can interact with apolipoprotein B (apoB) and high-density lipoprotein (HDL) (Scuruchi et al., 2020). In addition, decorin, which is a small CS/DSPG, interacts with type I collagen accumulated in atherosclerotic lesions and binds to LDL to enhance the retention of lipoproteins in the arterial wall (Pentikainen et al., 1997). There is also direct evidence that CS/DSPGs are involved in arteriosclerosis, the absence of chondroitin sulfate N-acetylgalactosaminyltransferase-2 can reduce lipoprotein retention in the early stage of atherosclerosis and reduce the migration of aortic smooth muscle cells (Adhikara et al., 2019). CSPG binds to lipoproteins mainly through its GAG chain. The accumulation of the LDL-GAG complex triggers a local inflammatory response to further promote the development of atherosclerosis (Nakashima et al., 2008).

The length of the CS chain and the sulfation modification mode are the main factors that affect the interaction between LDL and CSPG. The interaction between chondroitin-6-sulfate and LDL plays a vital role in atherosclerotic diseases. The increase in chondroitin-6-sulfate in early lesions of atherosclerosis leads to the accumulation, oxidation and hydrolysis of LDL and promotes the development of atherosclerosis (Karangelis et al., 2010; Cilpa et al., 2011).

### Other Diseases

In addition to the abovementioned physiological and pathological processes, CS/DS also plays a key regulatory role in inflammation, autoimmune diseases, genetic diseases, kidney diseases and other diseases. Studies have shown that CS/DSPGs promote the inflammatory response by binding immune receptors such as Toll-like receptors, selectin, CD44 and β1 integrin, which helps drive the development of traumatic brain/spinal cord injury and multiple sclerosis (Stephenson and Yong, 2018). The overexpression of chondroitin-6-sulfate in endothelial cells, which results in an imbalance of the chondroitin-6-sulfate and chondroitin-4-sulfate ratio, may be the main cause of chronic inflammatory diseases of the skin, such as skin lupus erythematosus and dermatomyositis (DM) (Kim and Werth, 2011). Defects in the core proteins of CS/DSPGs or mutations in CS/DS glycosyltransferases, epimerases, and sulfotransferases lead to a number of diseases, including congenital corneal stromal dystrophy, Meester-Loeys syndrome, and connective tissue diseases (Mizumoto et al., 2017; Kosho et al., 2019; Lautrup et al., 2020). The expression of CS/DS and DSPG is increased in a variety of fibrotic kidney diseases, including interstitial fibrosis, diabetic nephropathy, mesangial sclerosis and nephrosclerosis. Studies have shown that DSPGs regulate the formation of collagen fibrils in the body, so they play a role in kidney disease (Joladarashi et al., 2011; Lensen et al., 2015). In addition, CS/DS can also promote wound healing and improve osteoarthritis (Islam et al., 2020).

As mentioned above, CS/DS plays an important regulatory role in many physiological and pathological processes. An accurate understanding of the CS/DS mechanism in these processes will help in developing CS/DS-based polysaccharide drugs, along with new therapies and applications. The rapid progress of analytical methods will promote the decryption of the molecular basis of CS/DS-protein interactions. The following section will introduce the analytical methods used for characterizing GAG-protein interactions in recent years and their applications in CS/DS-proteins.

## Analytical Tools and Approaches for Characterization of GAG-Protein Interactions

**FIGURE 4:**
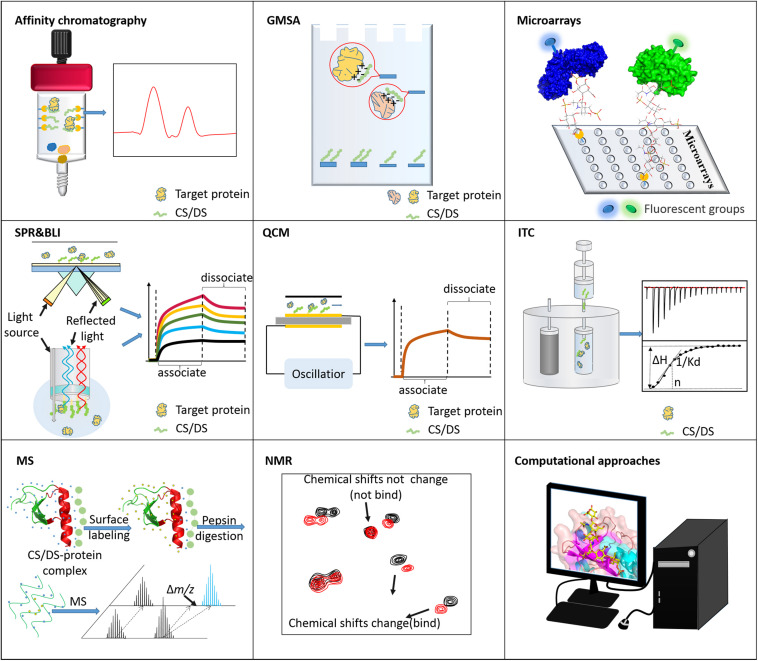
Tools for analyzing CS/DS-protein interactions. Affinity chromatography is usually used to isolate binding molecules or fragments. GMSA and microarrays are used to screen the CS/DS or proteins that bind to each other. ITC, SPR, BLI, and QCM can measure the binding strength between CS/DS and proteins. MS and NMR are applied to characterize the structure/sequence of binding motifs. Computational approaches are powerful for simulating the binding postures of these two types of biomolecules.

At present, many analytical methods for revealing the molecular mechanism and binding motifs of GAG-protein complexes have been developed, including affinity methods, MS, NMR, and computational approaches. The following reviews the progress and applications of various analytical methods in the analysis of CS/DS-protein complexes. Modern analytical tools that can be used to characterize the binding between CS/DS and proteins are categorized into different groups based on their purposes and summarized in Figure 4.

### Affinity Approaches

Many affinity-based analytical techniques and tools are used to explore the role of biomolecular interactions in physiological and pathological phenomena, including affinity chromatography, gel mobility shift assays (GMSA), isothermal titration calorimetry (ITC), biosensor-based surface plasmon resonance (SPR), biolayer interferometry (BLI), and quartz crystal microbalance (QCM) techniques. In research in recent decades, the above methods have not only been used to prove interactions between GAGs and proteins but have also been used to screen the interacting sequences of GAGs and proteins.

#### Affinity Chromatography

Affinity chromatography is a method used to separate interesting GAGs or interesting proteins from a mixture based on the highly specific macromolecular binding between GAG and the target protein. This method generally involves coupling interesting proteins or interesting GAGs to agarose and coating them onto affinity columns (Maimone and Tollefsen, 1990; Tollefsen, 1992; Barkalow and Schwarzbauer, 1994). Binding is usually carried out under physiological conditions, and then the target binding substance is eluted with increasing NaCl concentrations. GAGs are usually depolymerized into oligosaccharides to determine the binding domain of GAGs. For example, through affinity chromatography, Maimone and Tollefsen (1990) screened the DS hexasaccharide [IdoA(2S)-GalNAc(4S)-IdoA(2S)-GalNAc(4S)-IdoA(2S)-ATalR(4S)] with high affinity to heparin cofactor II (HCII). Bao et al. (2005) determined that under physiological salt concentrations, octasaccharide is the smallest CS/DS oligosaccharide that can interact with PTN. Similarly, site-directed amino acid mutations can identify key protein binding sites (Bao et al., 2005). In addition, based on the specific interactions of GAG-proteins, affinity chromatography also separates target proteins. Liangsupree et al. (2019) used immobilized chondroitin-6-sulfate to quickly separate plasma LDL. Toledo et al. (2020) used immobilized VAR2CSA to screen CSPGs that can interact with it. However, due to its inherent low resolution, elucidating of the GAG binding domain obtained by affinity chromatography often requires further separation and characterization.

#### GMSA

GMSA can show different electrophoretic mobility rates according to the size of the analyte molecule and the nature and size of the charge. It has been successfully applied to the interaction between GAG and proteins. Generally, AMAC is used to label the reducing end of GAG, and the offset of the GAG-protein complex band and the free GAG band is displayed by fluorescence irradiation to determine the binding affinity (Lyon et al., 2004). GMSA can simultaneously compare the relative protein affinities/selectivities of different oligosaccharides. Deakin et al. (2009) revealed that the binding of HGF/scatter factor (SF) to GAG depends on the degree of sulfation and not the position of sulfation. In addition, GMSA can also be used to measure protein binding sites. Boren et al. (1998) conducted a site-directed mutagenesis experiment and identified the basic amino acid whose interaction site between LDL and chondroitin-4-sulfate is located between residues 3,359 and 3,369.

#### ITC

ITC can directly obtain the enthalpy, entropy, free energy (binding constant), and stoichiometry in a single analysis. The binding constant can reflect the binding ability of two molecules, so ITC is also used for GAG-protein interaction analysis. To verify the structure of the interaction between CS-A and the VAR2CSA protein, which is a member of the erythrocyte membrane protein-1 family of *Plasmodium falciparum*, Singh et al. (2008). mutated Lys1507 and Lys1510 in the domain DBL3X to Ala, and the results of ITC analysis showed that their binding to CSA was significantly weakened, thus proving the binding site of CS-A on VAR2CSA. This work provides a new target for the development of a malaria vaccine (Singh et al., 2008). ITC can also be used to characterize GAG binding motif characteristics. Nguyen et al. (2019) used ITC to characterize the interactions between heparin oligosaccharides of different lengths and interleukin (IL)-12 when studying the molecular mechanism by which heparin induces and regulates the biological activity of human interleukin-12. The results showed that only heparin molecules longer than octasaccharide could enhance the activity of IL-12, and the more sulfate groups there were, the stronger the interaction (Nguyen et al., 2019).

#### SPR, BLI, and QCM

SPR can directly and quantitatively analyze unlabeled molecular interactions in real time and has been successfully used for the biophysical characterization of glycosaminoglycan (GAG) protein interactions. BLI is an SPR alternative technology developed later with a higher throughput and flexibility. As an example of a study detecting the binding motif of GAG, Zhang et al. (2015) used heparin biosensors to map the interactions between tissue inhibitor of metalloproteinases-3 (TIMP-3) and heparin and other GAGs via surface plasmonic resonance spectroscopy. The results show that TIMP-3 is a heparin-binding protein. The competition surface plasmon resonance results show that the interaction between TIMP-3 and heparin is chain length-dependent, in which *N*-sulfo and 6-*O*-sulfo groups play a key role in the interaction. Of the other GAGs, only CS-E and CS-B exhibit strong combination with TIMP-3 (Zhang et al., 2015). In the binding motif of the detection protein, to further understand the potential of *Borrelia burgdorferi* surface-localized membrane protein 1 (Lmp1)-interacting host molecule and the Lmp1 region that may participate in this interaction, Yang et al. (2016) placed a variety of host extracellular ligands on the chip and flowed the three discrete domains (Lmp1N, Lmp1 M, and Lmp1C) of recombinant Lmp 1 through the chip. The SPR results show that Lmp1 M interacts with chondroitin-6-sulfate and promotes the adhesion of Lmp 1 to host cells (Yang et al., 2016).

Different from SPR and BLI, QCM is an acoustic biosensor that uses the piezoelectric effect. The number of molecules bound on the surface determines the frequency of the QCM crystal, so the kinetic rate constant [association (*k*_*a*_) and dissociation (*k*_*d*_) rate constants] and affinity between the ligand and the analyte can be evaluated. Therefore, QCM has also been successfully used to characterize the interaction between GAGs and proteins. D’Ulivo et al. (2010) used QCM to study the interaction between LDL and chondroitin-6-sulfate. Three peptides involved in the interaction with glycosaminoglycans were selected from apo-B and fixed to polystyrene and carboxyl sensor chips. Chondroitin-6-sulfate was injected as an analyte on the peptide coating surface, and the estimated dissociation constant indicated that the interaction occurred through the positive residues lysine and arginine of ApoB-100 (D’Ulivo et al., 2010).

#### Microarrays

The high sensitivity and high throughput has made glycan microarray a core technology for analyzing GAG-mediated biological events. It is suitable for comparing binding strengths between ligand oligosaccharides and searching for specific oligosaccharide ligands (Yamaguchi et al., 2006). The key step in the construction of glycan microarrays is to immobilize glycans on the solid phase by non-covalent or covalent methods. Of course, many immobilization methods have been discussed (Palma et al., 2014; Song et al., 2015; Gao et al., 2019). Some of the GAGs used for glycan microarrays are characteristic oligosaccharides prepared by enzymatic hydrolysis or chemical desulfurization of natural GAG. Yamaguchi et al. (2006) used chondroitinase ACI to degrade DS into a series of oligosaccharides to prepare neoglycolipid microarrays and studied the interactions with HGF/SF, RANTES, KGF/FGF-7 and HCII. The results show that HGF/SF, KGF/FGF-7 and HCII can preferentially bind to DS oligosaccharide fragments longer than 8-mers, while the binding of RANTES depends on the strength of the charge (Yamaguchi et al., 2006). Shipp and Hsieh-Wilson desulfurized natural sources of HP and CS at different positions to prepare oligosaccharide microarrays interacting with various growth factors, proving that members of the FGF family and various axon guide proteins have obvious vulcanization preferences (Shipp and Hsieh-Wilson, 2007). Other GAGs used to construct glycan microarrays come from chemical or enzymatic synthesis, which greatly increases the number of glycans available for analysis in glycan microarrays. Tully et al. (2006) reported the first example of synthesizing CS microarrays and identified the interaction between CS-E and tumor necrosis factor-α (TNF-α) for the first time. Chemical-enzymatic synthesis promotes the efficient preparation of glycans with specific lengths and sulfation patterns, for example, three homogeneous CS-E oligosaccharides, including CS-E heptasaccharide, CS-E tridecasaccharide and CS-E non-adecasaccharide, have been produced recently (Li et al., 2020). Adoption of these chemically-enzymatically synthesized CS structures into glycan microarray will help to expand its potential to reveal more GAG-mediated biological events.

### MS

MS techniques have been maturely applied to GAG sequence mapping, protein epitope mapping and GAG binding protein structural characterization due to their high sensitivity, tolerance to low-purity samples, and ability to characterize single amino acid/sugar residues and modifications. In recent years, the MS methods used to characterize GAG-protein complexes have mainly included surface-labeling MS, cross-linking MS, ion mobility (IM) MS, and the recently emerged native MS. Among them, surface-labeling MS, which is more mature, is used to characterize the CS/DS-protein case. The principle of surface labeling is that chemical probes preferentially modify the parts of biomolecules exposed to the solvent, and the amino acids buried in the folded protein core or interacting proteins are not labeled. Then, proteolysis and LC/MS/MS are used to monitor the labeling site and degree of the protein (Lossl et al., 2016). According to different labeling methods, surface labels can be divided into covalent labels and non-covalent labels. Hydroxyl radical footprinting (HRF) uses hydroxyl radicals to rapidly oxidize amino acid side chains (Maleknia and Downard, 2014), which is a covalent labeling method for characterizing GAG-protein complexes. Wang K. et al. (2020) used hydroxyl radical HRF to characterize the binding sites between CS and the protein VAR2CSA expressed by the parasite. Hydroxyl radicals were used to rapidly oxidize the recombinantly expressed DBL1-ID2 protein with and without CS. After treatment with chymotrypsin, LC-MS/MS was used to compare the oxidation differences of each peptide. Finally, it was found that the peptide with the largest redox is peptide 543-558, which proves that the surface of the DBL2 and Hb1 groove acts as a CS binding region (Wang K. et al., 2020). The irradiation conditions of HRF must be carefully controlled to avoid secondary modification of the protein. Hydrogen-deuterium exchange (HDX) on the peptide backbone is a method to characterize the non-covalent labeling of protein-ligand complexes (Gallagher and Hudgens, 2016). The protein-ligand interaction changes the HDX rate of protein in deuterium water, which can provide rich information about the dynamic structure of protein-ligand complexes, including GAG-protein complexes. Tommy Hofmann performed a HDX experiment with IL-8 with and without CS. Through MS detection, the H/D exchange information of the entire IL-8 sequence was obtained. In the presence of CS, a significant reduction in H/D exchange was observed in the C-terminal α-helix region (containing amino acids 70–77) and loop (containing amino acids 27–29), which is the binding site of CS (Hofmann et al., 2015). HDX-MS analyzes GAG-protein complexes at the physiological pH, temperature and salt concentration, so it is a very promising technique for characterizing GAG-protein interactions.

### NMR

NMR is one of the most commonly used and valuable analytical methods for studying GAG-protein interactions. Commonly used NMR methods include chemical shift perturbation (CSP), saturation transfer difference (STD), and the transferred nuclear Overhauser effect (trNOE) (Pomin and Wang, 2018). The essence of the CSP method is to identify GAG-binding residues by assuming that when GAG is in contact with a protein, the atoms belonging to GAG-binding residues will show greater changes in chemical shifts. The atomic chemical shift of proteins is usually obtained by ^15^N-edited heteronuclear single quantum coherence (^15^N-HSQC) spectra of ^15^N-labeled proteins titrated with different GAG oligosaccharide concentrations (Pomin, 2014). Based on this method, Deshauer et al. (2015) studied the interaction of the chemokine CCL5 with medium-sized CS. The ^15^N-HSQC spectrum of CS titration showed that in addition to the BBXB motif in the 40 s loop, the CCL5 dimer also has a CS binding epitope located in the N loop, including the R17, L19 and I15 residues (Deshauer et al., 2015). By comparing the NMR spectra of the saturated state (on-resonance) and unsaturated state (off-resonance) of the interaction between the protein and the ligand in the solution (Vignovich and Pomin, 2020), the STD method can determine how GAG binds to protein during the formation of the GAG-protein complex. Yu et al. (2014) used saturated STD NMR to characterize the interaction of a synthesized heparin octasaccharide with FGF-2 and FGF-10. According to the STD value, the octasaccharide chain contained 2-*O*-sulfate and *N*- sulfate groups that participated in the binding of FGF-2, while 2-*O*-sulfate and 6-*O*-sulfate specifically participated in the binding of FGF-10 (Yu et al., 2014). The trNOE NMR method can provide information about the conformational changes of GAG oligosaccharides when interacting with proteins by identifying the changes in the NOE signal in the ligand molecule induced by the protein. Kunze et al. (2014) used the trNOE NMR method to study the binding of heparin tetrasaccharide (ΔUA2S-GlcNS6S-IdoA2S-GlcNS6S) and IL-10. The NOESY and ROESY profiles show that in the absence of IL-10, the NOE/ROE signal of the GAG ligand is close to zero. When IL-10 is present, in addition to a single positive NOE between H2 and H3 of the non-reduced terminal disaccharide GlCNS6s, several strongly negative NOEs were observed in heparin tetroglycosis, suggesting an appropriate molecular interaction between IL-10 and heparin (Kunze et al., 2014). Additional cases of using CSP, STD, and trNOE analysis to determine the protein binding motifs of GAG-protein complexes have been reported by other groups (Bechara et al., 2013; Mobius et al., 2013; Gao et al., 2018; Penk et al., 2019). NMR is an irreplaceable technique for the analysis of GAG-protein complexes. However, NMR requires highly skilled operators, and relatively low sensitivity is the main disadvantage of NMR.

### Computational Approaches

The complexity and heterogeneity of natural GAGs limit the in-depth study of the structure of GAG-protein interactions using various analytical tools. The latest developments in computational tools and technologies have made significant progress in the field of GAG modeling, and as an alternative strategy, they have promoted the study of GAG-protein interactions. Of course, various calculation methods and techniques have been discussed (Almond, 2018; Sankaranarayanan et al., 2018). Raghuraman et al. (2006) developed a CVLS method using the genetic algorithm-based automatic docking program GOLD and built up a heparin library consisting of a total of 6,859 unique heparin hexasaccharide sequences. Based on this method, several high-affinity and high-specificity heparin sequences were identified by AT recognition, and the binding mode of heparin pentasaccharide was accurately predicted (Raghuraman et al., 2006). Later, they applied CVLS to the DS-HCII system and screened 16 highly specific hexasaccharides from among 192 possible DS hexasaccharide topologies. Among them, 13 topologies were predicted to bind to the heparin binding site of HCII in a new binding mode at a 60-degree angle relative to the D helix (Raghuraman et al., 2010). The above prediction results based on CVLS are consistent with existing experimental data. Rogers et al. (2011) combined the carbohydrate microarray method with computational modeling to clarify the CS-E-neurotrophin (NT)-tyrosine receptor kinase (Trk) interaction. A continuous CS-E binding site spans the NT-Trk complex, which provides a potential mechanism explaining how CS regulates the formation of the complex and the NT signaling pathway. Later, cell experiments proved that CS plays an active role in cell signal transduction by regulating the NT-Trk interaction (Rogers et al., 2011). The GAG-Dock method successfully predicted the binding modes and sites of CS-A, CS-D, CS-E and heparin hexasaccharide to the axon growth-related protein RPTPσ and Nogo receptor. Among these interactions, it is predicted that when heparin participates in the binding of RPTPσ, multiple sulfate groups are exposed to the solvent, which can bind to other RPTPσ, and all the sulfate groups of CS-E point to the GAG binding site of RPTPσ, which explains the opposite effect of CS-E and heparin on neurite growth when interacting with RPTPσ (Griffith et al., 2017). Of course, the use of computational tools to predict the GAG-protein binding mode and site has been adopted by an increasing number of research groups (Pichert et al., 2012; Namachivayam et al., 2015; Ryan et al., 2016). In fact, various computing tools and websites have been developed to make the operation simple, and non-computational researchers can also perform calculation predictions (Sankaranarayanan et al., 2018).

## Conclusion and Remarks

Similar to other GAGs, CS/DS is involved in a large number of biological processes. In the body, CS/DS directly or indirectly participates in a variety of physiological and pathological processes by interacting with a variety of protein ligands, such as growth factors, cell surface receptors, and adhesion molecules. The characterization of a wide range of CS/DS-protein interactions is essential for mapping the biological functions of CS/DS and finding new therapies that target specific CS/DS-protein interactions. Due to the complexity and heterogeneity of the CS/DS structure, the molecular basis of most CS/DS-protein interactions is still unclear. The rapid development of multiple analytical tools and analytical methods will facilitate uncovering more of the mystery behind CS/DS-protein interactions and provide a template for the development of novel therapeutics based on CS/DS-protein interactions.

## Author Contributions

Both authors contributed to the article and approved the submitted version.

## Conflict of Interest

The authors declare that the research was conducted in the absence of any commercial or financial relationships that could be construed as a potential conflict of interest.

## Publisher’s Note

All claims expressed in this article are solely those of the authors and do not necessarily represent those of their affiliated organizations, or those of the publisher, the editors and the reviewers. Any product that may be evaluated in this article, or claim that may be made by its manufacturer, is not guaranteed or endorsed by the publisher.
